# Neural-immune-cancer crosstalk in pancreatic cancer: mechanisms and clinical translation

**DOI:** 10.3389/fcell.2026.1828946

**Published:** 2026-04-17

**Authors:** Wenjun Meng, Ping Yao, Manting Wang, Xinyue Pan, Jingzhang He, Yan Tie, Qinqin He, Rujun Zheng

**Affiliations:** 1 Department of Pain Management, West China Hospital, Sichuan University, Chengdu, China; 2 Department of Biotherapy, Cancer Center, West China Hospital, Sichuan University, Chengdu, China; 3 West China School of Medicine, Sichuan University, Chengdu, China

**Keywords:** neural-immune-cancer crosstalk, neuro-immune axis, pancreatic cancer, pancreatic ductal adenocarcinoma, tumor microenvironment

## Abstract

Pancreatic cancer remains a persistently high mortality rate, with limited efficacy through traditional therapies, necessitating exploration of its pathogenesis from a new biological perspective. The tumor microenvironment plays a decisive role in the malignant progression of pancreatic cancer, and the nervous system, as a key component of the microenvironment, has an active and bidirectional interaction with tumor cells, known as the “neuro-tumor interaction.” Pancreatic ductal adenocarcinoma (PDAC), the most common type of pancreatic cancer, is highly rich in neural components. The neuro-tumor interaction not only drives the unique neural infiltration of PDAC but also profoundly affects tumor proliferation, invasion, metastasis, immune escape, and pain perception. Recent studies have revealed that tumor cells, Schwann cells, cancer-associated fibroblasts, and immune cells form a “perineural niche” through neurotrophic factors, chemotactic axes, cell adhesion/extracellular matrix remodeling, and neurotransmitters, driving tripartite neural-immune-cancer interaction and providing targets for new therapeutic interventions. This review systematically summarized the key molecular and cellular mechanisms of neural-immune-cancer interactions in pancreatic cancer and specifically discussed several translational strategies, including neurotrophic factor blockade targeting NGF/TrkA and GDNF/RET, myeloid cell reprogramming targeting CXCR2/CXCL to improve T cell infiltration, and potential combination strategies that combine neuromodulatory drugs (e.g., β-blockers or CRGP antagonists) with immune checkpoint inhibitors. These strategies have shown feasibility in preclinical studies or PDAC models and warrant further validation in stratified clinical trials.

## 1 Introduction

Pancreatic ductal adenocarcinoma (PDAC) is the most common type of pancreatic cancer and one of the most malignant solid tumors, with high mortality rate remaining, and limited effectiveness in traditional therapies (Stoop et al., 2025). Therefore, it is urgent to explore its pathogenesis from a new biological perspective. In recent years, the decisive role of the tumor microenvironment (TME) in the malignant evolution of PDAC has received increasing attention (Arnold et al., 2026). Among them, the nervous system, as a key component of the TME, has an active and bidirectional interaction with tumor cells, namely, “neuro-tumor interaction” (Wang et al., 2026). This interaction not only drives the unique neural infiltration phenomenon of PDAC, but also profoundly affects the proliferation, invasion, metastasis, immune escape and pain perception of the tumor (Hussain et al., 2025). Studies have shown that tumor cells can form synapse-like structures with neurons and remodel local neural circuits by releasing neurotransmitters, neurotrophic factors and other signaling molecules, thereby promoting their own growth and adaptation (Ren et al., 2025). Meanwhile, this interaction also constitutes a complex “neuro-immune axis.” Neural signals can regulate the phenotype and function of tumor-associated immune cells (such as T cells, macrophages, myeloid-derived suppressor cells, etc.), thereby affecting the anti-tumor immune response (Wang et al., 2026; Zhang C. et al., 2025). Understanding the complex signaling network among nerves, tumor cells and their immunity is crucial for developing breakthrough diagnostic and therapeutic methods. This review will systematically review the research on neural-immune-cancer crosstalk in PDAC, highlight its central role in tumor biology, and explore targeting this interface, especially its bidirectional association with cancer immunological response, to provide new theoretical basis and translational directions for developing more effective treatment strategies.

## 2 Cellular components and functional division of perineural niche in pancreatic cancer

The perineural niche refers to the specific microenvironment surrounding the peripheral nerves, composed of nerve cells, supporting cells, recruited inflammatory cells, altered extracellular matrix (ECM), blood vessels, and immune components (Chen et al., 2019). In the context of cancer, this microenvironment becomes a key site for the interaction between tumor cells and nerves, promoting the occurrence and development of perineural invasion (PNI) (Wang et al., 2021). The perineural niche of pancreatic cancer is a complex ecosystem in which the direct interaction between nerve cells and tumor cells is the core mechanism driving tumor progression. This interaction is mainly achieved through the infiltration of nerve axons into tumor tissue (i.e., neurogenesis) and the migration of tumor cells into nerve fibers, which is the biological process of PNI (Li et al., 2023).

### 2.1 Direct interaction between nerve cells and tumor cells

**FIGURE 1:**
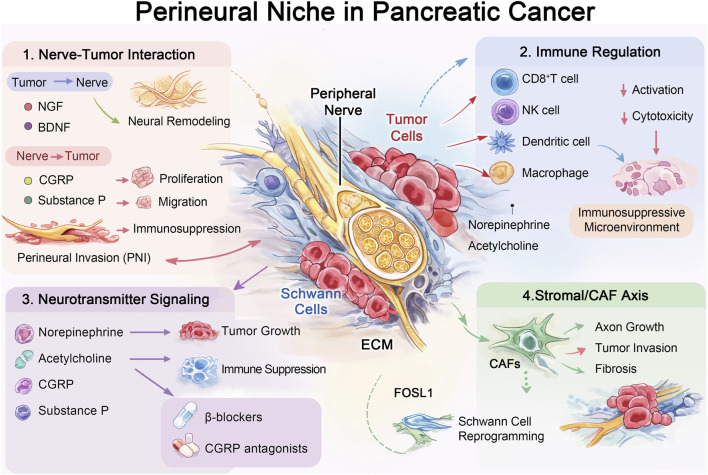
The perineural niche in pancreatic cancer represents a complex ecosystem composed of tumor cells, peripheral nerves, immune cells, stromal cells, and extracellular matrix components. Bidirectional interactions between tumor cells and nerves drive perineural invasion (PNI), while neurotransmitters and neuropeptides regulate immune suppression and tumor progression. Stromal components such as cancer-associated fibroblasts further remodel the niche through cytokine and extracellular matrix secretion. Targeting neuro-tumor signaling pathways may represent a promising therapeutic strategy.

In pancreatic cancer, PNI is a prominent feature, which is not only a pathway for tumor spread, but also creates a neural niche that supports tumor cell survival and invasion (Xu et al., 2025a). Nerve fibers act directly on pancreatic cancer cells in a paracrine manner by releasing neuropeptides, such as calcitonin gene-related peptide (CGRP), driving tumor growth and inducing immunosuppression (Xu M. et al., 2025). In addition, neurotrophic factors such as nerve growth factor (NGF) and brain-derived neurotrophic factor (BDNF) play a key role in the progression of pancreatic cancer, promoting neural remodeling and enhancing the migration and invasion capabilities of tumor cells (Xu et al., 2025a). This bidirectional signal transduction constitutes the “addiction” of tumors to nerves, providing a therapeutic opportunity to target the neuro-tumor interaction (Xu M. et al., 2025) (Figure 1).

### 2.2 The role and regulation of immune cells in the perineural niche

Immune cells in the perineural niche are a key bridge connecting the nervous system and tumor immune surveillance. Studies have shown that immune cells infiltrating the TME express a variety of neurotransmitter receptors, enabling them to directly respond to neural signals (Li et al., 2023). For example, in a gastric cancer model, acetylcholine (ACh) released by the vagus nerve can promote the production of immunosuppressive cytokines such as interleukin-22 (IL-22) by regulating cancer cell metabolism, thereby upregulating the expression of programmed death-ligand 1 (PD-L1) and enhancing tumor immune resistance (Liao et al., 2026). However, direct experimental evidence for PDAC is still limited. Since IL-22 is indeed highly expressed in PDAC and is associated with poor prognosis, the above gastric cancer model need to be validated in PDAC (Perusina et al., 2020). In pancreatic cancer, the interaction between the nervous and immune systems is equally profound. The sympathetic and parasympathetic nervous systems regulate the function of immune cells in the tumor microenvironment by releasing neurotransmitters such as norepinephrine, such as affecting the activation of dendritic cells, the cytotoxicity of natural killer (NK) cells and CD8^+^ T cells, and the polarization of myeloid cells, thereby establishing an immunosuppressive state that is conducive to tumor growth and metastasis (Xu N. et al., 2025). This neuro-immune axis dysregulation is one of the important mechanisms of tumor immune escape (Vanden Abeele and Salzet, 2025).

### 2.3 Synergy between stromal cells and the neuro-immune axis

Cancer-associated fibroblasts (CAFs) are abundant stromal components in the TME of pancreatic cancer, playing a synergistic role in the formation and function of the perineural niche. There is a dynamic interaction network between CAFs and nerve and immune cells (Wang et al., 2024). These cells jointly construct and reshape the neural niche by secreting cytokines, chemokines and ECM components. For example, CAFs can secrete neurotrophic factors or express neural guidance molecules to promote the growth of nerve axons and the PNI of PDAC tumor cells (Li et al., 2025). Meanwhile, neural signals can also feedback regulate the activation and function of CAFs. This complex interaction involving stromal cells, cytokines and neurotrophic factors significantly promotes the characteristic fibrosis and neural invasion process of PDAC. In addition, transcription factors such as FOS-like antigen 1 (FOSL1) have been identified as the core regulatory hub connecting the tumor and the nervous system (Maietta et al., 2025). It promotes neural remodeling and TME reprogramming through mechanisms such as regulating Schwann cell reprogramming and chemokine network construction, reflecting the central role of stromal cells in the neural-immune-cancer synergistic network (Pu et al., 2025).

### 2.4 Signaling networks of neurotransmitters and neuropeptides

Neurotransmitters and neuropeptides are key signaling molecules that mediate intercellular communication within the perineural niche (Zhang et al., 2026). In addition to being released by neurons, these molecules can also be secreted by cancer cells and immune cells in the TME, forming an autocrine and paracrine signaling network (Li et al., 2023). In pancreatic cancer, important signaling molecules include norepinephrine, acetylcholine, substance P, and CGRP (Xu N. et al., 2025). These molecules broadly regulate the proliferation, survival, and migration of tumor cells by binding to their respective receptors and profoundly affect the function of immune cells. For example, norepinephrine can inhibit anti-tumor immune responses through the β-adrenergic receptor signaling pathway (Liu et al., 2025); while CGRP has been shown to be a key neurotrophic factor driving tumor growth and immunosuppression (Xu M. et al., 2025). Targeting these neural signaling pathways, such as using β-receptor blockers or CGRP receptor antagonists, has become an emerging therapeutic strategy for reversing immunosuppression and enhancing anti-tumor immunity.

## 3 Key molecules and signaling of neural-immune-cancer crosstalk in pancreatic cancer

### 3.1 Neurotrophic factor axis

The neurotrophic factor axis, particularly the interaction between nerve growth factor (NGF) and its high-affinity receptor TrkA, plays a central role in the neural-immune-cancer interactions of pancreatic cancer (Wang Y. et al., 2025). NGF is secreted by various cells in the tumor microenvironment and mediates not only the neurochemotaxis of cancer cells but is also closely associated with cancer-related pain (Wang K. et al., 2025). Upon binding to TrkA, NGF activates key downstream MAPK and PI3K signaling pathways, the activation of which directly promotes the proliferation, survival, and invasion of tumor cells (Wang Y. et al., 2025). More importantly, this signaling axis upregulates the expression of matrix metalloproteinases (MMPs), which create conditions for tumor cell invasion and PNI by degrading physical barriers such as the ECM and nerve sheaths. Therefore, targeting the NGF/TrkA axis (e.g., using the Trk inhibitor larotrectinib) is considered a promising therapeutic strategy aimed at blocking tumor-neural communication and overcoming treatment resistance (Xie et al., 2024).

### 3.2 GDNF family ligands and related receptors

Glial cell line-derived neurotrophic factor (GDNF) family ligands, such as Artemin, are primarily produced by peripheral nerves and ganglia and play a crucial role in the neural guidance of pancreatic cancer (Cavel et al., 2012). Experimental studies have demonstrated that pancreatic cancer cells exposed to GDNF exhibit significantly enhanced invasion and chemotactic movement along neural structures, suggesting that GDNF acts as a potent neurotropic factor facilitating PNI (Gil et al., 2010). Tumor cells are chemically attracted to the perineurium by expressing receptors like RET for these ligands, a process that is a key step in PNI (Wang Y. et al., 2025). In addition, the binding of GDNF to the RET/GFRα1 complex can activate downstream signaling pathways such as PI3K/AKT and MAPK, which enhance tumor cell survival and motility within the perineural niche (Airaksinen and Saarma, 2002). Activation of GDNF related signaling is closely associated with the invasive, survival, and metastatic potential of tumor cells, making it a potential target for translational therapy.

### 3.3 Chemotaxis and adhesion axis

The chemical gradient formed by chemokines and their receptors is the core mechanism driving the directional migration of pancreatic cancer cells to the nerve. Among them, the CXCL12/CXCR4 axis has been extensively studied, and the activation of CXCR4 can regulate a variety of processes such as cell proliferation, survival, migration and angiogenesis (Ghasemi and Ghasemi, 2022). In addition, the CCL2/CCR2 and CX3CL1/CX3CR1 axes also play a role in establishing the chemical gradient in the TME and recruiting immune cells and cancer cells (Liang et al., 2026). After cancer cells approach the nerve, specific adhesion molecules mediate strong tumor-nerve adhesion. For example, the interaction between MUC1 and myelin-associated glycoprotein (MAG) and L1 cell adhesion molecule (L1CAM) significantly enhance the adhesion between cancer cells and nerve components, laying the foundation for subsequent invasion and nerve encapsulation (Wang Y. et al., 2025). The synergistic effect of these chemokine and adhesion mechanisms jointly promotes the occurrence of PNI.

### 3.4 Extracellular matrix (ECM) and protease

ECM remodeling and protease activity are key to pancreatic cancer cells crossing physical barriers such as nerve sheaths. ECM components such as Tenascin-C and fibronectin not only provide adhesion sites for migrating tumor cells, but their altered expression patterns also create a pro-invasive microenvironment (Furuhashi et al., 2023). Meanwhile, the matrix metalloproteinase (MMP) family is significantly upregulated, and its activity is regulated by neurotrophic factors such as the NGF/TrkA signaling axis (Kolli and Kumar, 2026; Xu et al., 2025b). MMPs effectively reduce the physical barrier resistance around nerves by synergistically degrading collagen and basement membrane components in the ECM. The synergistic effect of ECM proteins and proteases opens up pathways for tumor cells, enabling them to cross the tough nerve sheath and achieve PNI.

### 3.5 Inflammation/transcription axis

Inflammatory signaling and transcriptional regulatory networks profoundly shape the neuroinvasive microenvironment of pancreatic cancer (Marcadis et al., 2023). Transforming growth factor-β (TGFβ) through the SMAD signaling pathway, interleukin-6 (IL-6) through the activation of STAT3 transcription factor, and nuclear factor-κB (NF-κB) pathway jointly participate in the reprogramming of Schwann cells, transforming them into a phenotype that supports tumor growth (Sharawy, 2022). These pathways also drive the formation of an immunosuppressive microenvironment, helping tumors achieve immune escape by regulating cytokine expression and immune cell function. Given the central role of JAK/STAT and TGFβ signaling in tumor progression and immune regulation, their inhibitors are considered potential candidates for combination therapy strategies. For example, targeting the IL-6/JAK2/STAT3 pathway has been shown to be associated with the progression of various cancers, and its inhibition may simultaneously affect tumor cell behavior and the immune microenvironment (Zhang et al., 2018; Huang et al., 2022).

### 3.6 Axial guidance and neural plasticity

**FIGURE 2:**
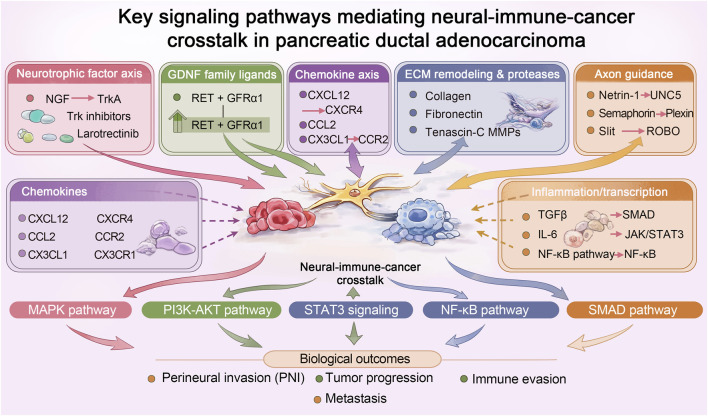
Molecular signaling networks mediating neural–immune–cancer crosstalk in pancreatic cancer. Neurotrophic factors, chemokines, inflammatory signaling, extracellular matrix remodeling, and axon guidance pathways coordinate the interactions between tumor cells, nerves, and immune cells within the perineural niche. These pathways activate downstream signaling cascades, including MAPK, PI3K-AKT, STAT3, NF-κB, and SMAD signaling, ultimately promoting perineural invasion, tumor progression, immune suppression, and metastasis in pancreatic ductal adenocarcinoma.

Axonal guidance molecules play a crucial role in the direction of neural remodeling and PNI in pancreatic cancer. Netrin-1 and its receptor UNC5, as well as the Semaphorin (SEMA) and Plexin/ROBO receptor families, provide repulsive or attractive signals that guide the growth of cancer cells and neural synapses, and are involved in tumor-associated neural plasticity processes (Haidar et al., 2025; Meng et al., 2024; Tsuboi et al., 2025). Dysregulation of these molecular pathways is an important mechanism by which the tumor microenvironment “rewires” to promote invasive growth (Wang Y. et al., 2025). With the development of spatial transcriptomics and single-cell sequencing technologies, scientists have been able to resolve the cellular composition and transcriptional program of PNI regions at unprecedented resolution. These technologies can reveal specific gene expression features and intercellular communication networks associated with PNIs, providing valuable clues for the discovery of new diagnostic biomarkers and the development of precision therapeutic targets targeting the tumor-neural interface (Figure 2).

### 3.7 Neural regulation of immune cell subsets in PDAC

Immune cell responses to neurotrophic factors or neurotransmitters can be achieved through two mechanisms: (a) immune cells themselves express corresponding receptors (e.g., immune cells can express β-adrenergic receptors or certain neuropeptide receptor components); (b) neural signals firstly reprogram stromal cells (CAF, Schwann cells, tumor cells), causing them to alter the secretion of chemokines/cytokines and metabolites, thereby indirectly regulating the recruitment, polarization, and function of immune cells. Review evidence supports the coexistence of these two mechanisms, but there is significant heterogeneity in the “direct vs. indirect” weighting among different tumors and different immune cells (Xu et al., 2025a; Amit et al., 2025). Specific subtypes within PDAC need to be differentiated based on single-cell, spatial transcriptomics and functional experimental evidence. Table 1 summarizes the mechanisms and evidence for the regulation of immune cell subsets in PDAC by various neurotrophic factors.

**TABLE 1:** The mechanisms and evidence for the regulation of immune cell subsets in PDAC.

Immune cell subset	Main neural factors/Neurotransmitters	Main regulatory mechanisms (direct or indirect)	Direct evidence in PDAC	References
TAM (tumor-associated macrophages)	Norepinephrine (NE); neurotrophic factors (indirect)	β-adrenergic receptor (β-AR)-mediated polarization (direct); alteration of population composition via chemokine changes (indirect)	PDAC mouse evidence links chronic stress/NE to immunosuppression; chemokine axes (e.g., CXCR2) affect myeloid cell recruitment and thereby influence TAM composition and function	Partecke et al. (2016), Steele et al. (2016)
CD8^+^ cytotoxic T cells	Norepinephrine (NE); calcitonin gene-related peptide (CGRP) (inhibitory)	Suppression of effector function and decreased IFN-γ/granzyme expression (direct); indirect suppression via inhibition of dendritic cell (DC) maturation or antigen presentation	PDAC models show that relieving immunosuppressive constraints (e.g., Treg or myeloid suppression) can restore CD8^+^ IFN-γ expression, indicating neural–immune signals affect CD8^+^ function	Partecke et al. (2016), Jang et al. (2017)
Regulatory T cells (Tregs)	Sympathetic signals (NE), etc.	Recruitment/maintenance (indirect) and possible direct modulation of function	Chronic stress increases intratumoral Treg abundance in mouse models; Tregs are associated with immunosuppression in PDAC.	Partecke et al. (2016), Jang et al. (2017)
MDSCs/neutrophils (myeloid-derived suppressor cells/granulocytic cells)	CXCL/CXCR2 family (stroma-induced); NE may indirectly promote	Chemokine-mediated recruitment (indirect) → suppression of T cells (indirect)	CXCR2 inhibition reduces MDSC recruitment and improves responses to immunotherapy in PDAC mouse models	Steele et al. (2016), Tcyganov et al. (2018)
Dendritic cells (DCs)	CGRP; norepinephrine (NE)	Direct inhibition of maturation/co-stimulatory molecule expression (direct); induction of metabolic changes (e.g., IDO) that promote tolerogenic phenotype (indirect)	Tumor-associated DCs in PDAC often exhibit a tolerogenic phenotype; Treg-DC interactions have been demonstrated to limit DC immunogenicity	Amit et al. (2025), Jang et al. (2017)

## 4 Clinical relevance and translatable strategies

### 4.1 PNI is a poor prognostic factor for PDAC and is associated with pain

PNI in PDAC has been widely recognized as a key prognostic factor. The presence of PNI is closely associated with more aggressive biological behavior of the tumor, including a higher incidence of lymph node metastasis, an increased risk of early recurrence, and significantly reduced recurrence-free survival (RFS) and disease-specific survival (DSS) (Nozzoli et al., 2024). Further studies have shown that the severity of PNI is positively correlated with the extent of lymphatic and vascular invasion, suggesting an intrinsic link between nerve invasion and the mechanism of tumor spread through other pathways (Wakiya et al., 2021). In addition to promoting tumor progression, PNI is also an important driver of pain in PDAC patients. Tumor cell invasion of nerves activates nociceptive neurons, leading to neurogenic inflammation and central sensitization, resulting in significant cancer pain. Preclinical models have shown that as PDAC tumors grow and PNI develops, mice exhibit progressively worsening abdominal mechanical hyperalgesia, arched back behavior, and anxiety-like behavior, which are directly related to histologically observed PNI (Hua et al., 2025). Therefore, PNI is not only a pathological indicator, but also a key bridge connecting local tumor invasion with systemic symptoms (such as pain), profoundly affecting patients’ quality of life and overall prognosis.

### 4.2 Translational therapeutic strategies targeting the neural-immune-cancer interactions in PDAC

Transformable therapeutic strategies targeting the neural-immune-cancer interaction in PDAC are becoming a research hotspot. On the one hand, direct targeting of neuro-tumor communication is a key direction, including using anti-NGF/Trk inhibitors (such as larotrectinib) to block neurotrophic factor signaling, and inhibiting the GDNF/RET pathway, which has been shown to drive PDAC PNI and metastasis by activating RAC2 GTPase through phosphorylation of MUC21 (Chen et al., 2024). In addition, targeting the chemokine receptor CXCR4 can interfere with the chemotactic migration of tumor cells along nerves (Chen et al., 2026; Sun et al., 2026). On the other hand, regulating systemic neural signals also shows potential, for example, using β-receptor blockers (such as propranolol) to inhibit the pro-tumorigenic effects of the sympathetic nervous system, although its effects in pancreatic cancer are heterogeneous and should be used with caution (Zhang F. et al., 2025). The heterogeneous effects in PDAC, possibly due to factors such as the β-receptor selectivity of different drugs (non-selective propranolol versus selective beta1 inhibitors), timing of administration (pre-diagnosis/perioperative/combined with chemotherapy), dosage and duration of use, as well as confounding bias in population studies and differences in tumor molecular subtypes (Udumyan et al., 2017). PDAC stress models in mice have shown that propranolol can partially reverse stress-related immunosuppression and slow tumor growth, while retrospective population studies have yielded inconsistent results, suggesting that future clinical studies should be stratified by drug class, timing of administration, and patient subgroup (Partecke et al., 2016; Pantziarka et al., 2016).

For pain relief, local nerve blocks can not only effectively control symptoms, but may also alter the TME in the short term. In diagnosis and monitoring, liquid biopsy techniques, particularly exosome (EV)-based analysis, offer a powerful, non-invasive tool. Specific molecules carried in exosomes, such as the neurotrophic factor receptor p75NTR, have the potential to serve as candidate biomarkers reflecting the neuroinvasive state of tumors and disease progression, thereby enabling earlier intervention and efficacy assessment (Grinspan and Villanueva, 2022; Wong et al., 2025).

### 4.3 Translating neural-immune-cancer target into clinical practice presents multiple challenges

Currently, the assessment of PNI mainly relies on qualitative or semi-quantitative descriptions of pathology, lacking standardized quantitative indicators (such as precise neuroinvasion counts and depth scoring systems), which hinders accurate risk stratification and efficacy comparison (Nozzoli et al., 2024). To overcome these obstacles, future development depends on two core strategies: first, developing reliable biomarkers based on multi-omics (such as transcriptomics and proteomics) for fine stratification of patients and identifying the subgroups most likely to benefit from specific neurotargeted therapies; and second, designing early to mid-stage clinical trials driven by safety and clear clinical endpoints (such as pain relief and progression-free survival) (Figgett et al., 2019). Adaptive trial design, integration of real-world evidence, and use of computational models (such as quantitative systems pharmacology) for virtual patient simulation and trial optimization are crucial for successfully advancing novel therapies in such a complex disease context (Sun et al., 2025).

## 5 Unresolved issues and future directions

The neural-immune-tumor interactions in pancreatic cancer are complex, with different neural subtypes (sympathetic, parasympathetic, and sensory) playing heterogeneous roles in tumorigenesis, progression, and PNI. Their relative contributions and therapeutic windows need to be clarified. Sympathetic activity is associated with physiological or psychological stress, and tumor development and diagnosis themselves can induce such stress responses (Scheff and Saloman, 2021). Targeting stress responses, such as inhibiting sympathetic activity or activating parasympathetic activity, has been shown to drive the activation of effector T cells within the tumor and inhibit myeloid-derived suppressor cells (Scheff and Saloman, 2021). However, neural effects are significantly tumor-type specific; for example, β-blockers have shown a protective association in breast cancer and melanoma, but may have detrimental effects in pancreatic and head and neck cancers (Zhang F. et al., 2025). This suggests that sympathetic signaling may have a unique pro-cancer effect in pancreatic cancer. Meanwhile, the role of sensory nerves in regulating tumor growth and metastasis is receiving increasing attention, as they may exert their effects in a tumor-type specific manner by promoting or inhibiting immunosuppression. Therefore, future research needs to utilize technologies such as single-cell sequencing and spatial transcriptomics to accurately analyze the spatial distribution, molecular characteristics, and dynamic interaction networks of different neural subtypes with immune cells and tumor cells in the pancreatic cancer microenvironment, in order to identify specific neural targets with therapeutic potential and determine the optimal timing for intervention.

Schwann cells (SCs) play a key role in pancreatic cancer PNI, and the molecular mechanisms by which they transform from a “repairing” phenotype that supports neurogenesis to a “tumor helper” phenotype that promotes tumor invasion are unclear (Chen M. et al., 2025). Studies have shown that there is complex bidirectional communication between tumor cells and SCs (Chen K. et al., 2025). For example, in distal cholangiocarcinoma, lactate in the hypoxic TME can induce GFAP-positive dedifferentiation in SCs, which in turn promotes cancer cell invasion by upregulating HMGB1 (Zu et al., 2025). This phenotypic shift may involve multiple molecular triggers, including NGF signaling, extracellular vesicle-mediated molecular transmission (e.g., O-GlcNAcase) (Zhao et al., 2025), and epigenetic reprogramming. SCs themselves have significant epigenetic plasticity, which enables them to change their functional state in response to microenvironment signals (Ebrahim et al., 2025). Future research needs to systematically identify key signaling pathways (such as NGF/TrkA, RhoA/JNK, etc.) and epigenetic regulators (such as DNA methylation, histone modification, and non-coding RNA) that drive SC phenotypic transformation (Qian et al., 2024; Liu et al., 2023). More importantly, it is necessary to assess whether this “tumor helper” phenotype is reversible. Preclinical models have shown that targeting the axis of SC-tumor cell interaction (such as the HMGB1-RAGE axis or the SRC-1/STAT1/MMP12 axis) can inhibit PNI progression (Zhao et al., 2025; Cheng et al., 2025). This provides a theoretical basis for “reprogramming” SCs back to a repair state or inhibiting their tumor-promoting function through pharmacological or bioelectronic means, which is a highly promising therapeutic direction.

Currently, there is a lack of standardized quantitative systems for the research and clinical evaluation of PNI, which hinders the accurate judgment of the prognostic value of PNI, the comparison between different research results, and the effective evaluation of new therapies. At the histological level, there are differences in the definition of PNI (such as the proportion of nerve circumference surrounded by tumor cells) and the quantification methods (such as simply recording the presence or absence, or counting the number of invaded nerves or assessing the extent of invasion), which leads to controversy over its value as an independent prognostic predictor (Niu et al., 2022; Wu et al., 2020). There are currently a variety of PNI models, such as the sciatic nerve injection model, the whisker pad model, and the tumor-dorsal root ganglion co-culture system (Cheng et al., 2025; Zhang M. et al., 2025; de Lima et al., 2023). In the future, we should promote the standardization of these models and use *in vitro* systems such as organoid co-culture and microfluidic chips to conduct in-depth research on the cellular and molecular mechanisms of PNI under controlled conditions, providing a reliable platform for drug screening and mechanism research.

## 6 Conclusion

The complex neural-immune-cancer network in PDAC has progressed from early pathological observations to a recognized core biological mechanism. Evidence indicates that interactions among tumor cells, nerves, immune cells, and stromal components-mediated by neurotrophic factors, chemotactic signaling, ECM remodeling, inflammation, and neurotransmitters, play a critical role in tumor progression and therapeutic resistance. Future studies should focus on clarifying the spatiotemporal dynamics and cellular origins of these pathways to better understand PDAC heterogeneity. For clinical translation, pathways with strong mechanistic evidence, such as NGF/TrkA, GDNF/RET, CXCL12/CXCR4, and Netrin-1/DCC, may serve as promising therapeutic targets, although their dual roles in tumor biology and tissue homeostasis warrant careful evaluation. Successful translation will depend on biomarker-guided patient stratification and rational combination strategies integrating neurotargeted therapies with chemotherapy, immunotherapy, or radiotherapy. Ultimately, mechanism-based targeting of neural-immune-cancer interactions may move beyond symptom control to improve tumor control and survival in PDAC patients.

## References

[B1] Airaksinen M. S. Saarma M. (2002). The gdnf family: signalling, biological functions and therapeutic value. Nat. Rev. Neurosci. 3 (5), 383–394. 10.1038/nrn812 11988777

[B2] Amit M. Eichwald T. Roger A. Anderson J. Chang A. Vermeer P. D. (2025). Neuro-immune cross-talk in cancer. Nat. Rev. Cancer 25 (8), 573–589. 10.1038/s41568-025-00831-w 40523971 PMC13142818

[B3] Arnold F. Del Vecchio A. Hussain Z. Sherman M. H. (2026). Heterocellular crosstalk and architecture of the pancreatic tumour microenvironment. Nat. Rev. Cancer 26 (4), 285–304. 10.1038/s41568-025-00905-9 41545706

[B4] Cavel O. Shomron O. Shabtay A. Vital J. Trejo-Leider L. Weizman N. (2012). Endoneurial macrophages induce perineural invasion of pancreatic cancer cells by secretion of gdnf and activation of ret tyrosine kinase receptor. Cancer Res. 72 (22), 5733–5743. 10.1158/0008-5472.Can-12-0764 22971345

[B5] Chen S. H. Zhang B. Y. Zhou B. Zhu C. Z. Sun L. Q. Feng Y. J. (2019). Perineural invasion of cancer: a complex crosstalk between cells and molecules in the perineural niche. Am. J. Cancer Res. 9 (1), 1–21. 30755808 PMC6356921

[B6] Chen Y. Zhang W. Zeng Y. Yang P. Li Y. Liang X. (2024). Gdnf-induced phosphorylation of Muc21 promotes pancreatic cancer perineural invasion and metastasis by activating Rac2 gtpase. Oncogene 43 (34), 2564–2577. 10.1038/s41388-024-03102-4 39020072

[B7] Chen M. M. Gao Q. Ning H. Chen K. Gao Y. Yu M. (2025). Integrated single-cell and spatial transcriptomics uncover distinct cellular subtypes involved in neural invasion in pancreatic cancer. Cancer Cell 43 (9), 1656–1676.e10. 10.1016/j.ccell.2025.06.020 40680743

[B8] Chen K. Chen Z. Wang J. Zhou M. Liu Y. Xu B. (2025b). Single-cell sequencing unravels pancreatic cancer: novel technologies reveal novel aspects of cellular heterogeneity and inform therapeutic strategies. Biomedicines 13 (12), 3024. 10.3390/biomedicines13123024 41463034 PMC12730243

[B9] Chen K. Ma Y. Huang L. Wu P. Kung H. C. Yang B. (2026). Complement-secreting cafs are associated with better prognosis in pancreatic cancer: single-cell multiomics. Gut. 10.1136/gutjnl-2025-335683 41534892 PMC13311983

[B10] Cheng K. Liu L. Gong M. Ji Y. Bai C. Guo X. (2025). Steroid receptor Coactivator-1 drives tumor-associated macrophage reprogramming by mediating Mmp12 transcription in pancreatic cancer perineural invasion. Adv. Sci. (Weinh) 12 (45), e16575. 10.1002/advs.202416575 40985319 PMC12677693

[B11] de Lima P. O. Broit N. Huang J. D. Lim J. H. Gardiner D. J. Brown I. S. (2023). Development of an *in vivo* murine model of perineural invasion and spread of cutaneous squamous cell carcinoma of the head and neck. Front. Oncol. 13, 1231104. 10.3389/fonc.2023.1231104 37746297 PMC10513369

[B12] Ebrahim N. A. A. Soliman S. M. A. Othman M. O. Tahoun N. S. (2025). Molecular mechanisms and clinical significance of perineural invasion in malignancies: the pivotal role of tumor-associated schwann cells in cancer progression and metastasis. Med. Oncol. 42 (5), 171. 10.1007/s12032-025-02729-x 40259163

[B13] Figgett W. A. Monaghan K. Ng M. Alhamdoosh M. Maraskovsky E. Wilson N. J. (2019). Machine learning applied to whole-blood Rna-Sequencing data uncovers distinct subsets of patients with systemic lupus erythematosus. Clin. Transl. Immunol. 8 (12), e01093. 10.1002/cti2.1093 31921420 PMC6946916

[B14] Furuhashi S. Morita Y. Matsumoto A. Ida S. Muraki R. Kitajima R. (2023). Tenascin C in pancreatic cancer-associated fibroblasts enhances epithelial mesenchymal transition and is associated with resistance to immune checkpoint inhibitor. Am. J. Cancer Res. 13 (11), 5641–5655. 38058842 PMC10695794

[B15] Ghasemi K. Ghasemi K. (2022). Msx-122: is an effective small molecule Cxcr4 antagonist in cancer therapy? Int. Immunopharmacol. 108, 108863. 10.1016/j.intimp.2022.108863 35623288

[B16] Gil Z. Cavel O. Kelly K. Brader P. Rein A. Gao S. P. (2010). Paracrine regulation of pancreatic cancer cell invasion by peripheral nerves. J. Natl. Cancer Inst. 102 (2), 107–118. 10.1093/jnci/djp456 20068194 PMC2911041

[B17] Grinspan L. T. Villanueva A. (2022). Biomarker development using liquid biopsy in hepatocellular carcinoma. Semin. Liver Dis. 42 (2), 188–201. 10.1055/s-0042-1748924 35738257

[B18] Haidar H. Bellon A. Sleiman K. Hocine M. Rama N. Gadot N. (2025). Neural function of Netrin-1 in precancerous lesions of the pancreas. Nat. Commun. 16 (1), 7094. 10.1038/s41467-025-62299-4 40753071 PMC12318043

[B19] Hua B. Huang X. Chen L. Li Y. Lu D. Wang D. (2025). Slc26a9 promotes the perineural invasion of pancreatic cancer in mice. Biochem. Biophys. Res. Commun. 791, 152952. 10.1016/j.bbrc.2025.152952 41232379

[B20] Huang B. Lang X. Li X. (2022). The role of Il-6/Jak2/Stat3 signaling pathway in cancers. Front. Oncol. 12, 1023177. 10.3389/fonc.2022.1023177 36591515 PMC9800921

[B21] Hussain M. S. Maqbool M. Agrawal M. Rana A. J. Sultana A. Sulthana N. (2025). Exploring the neurobiological mechanisms of cancer growth. Curr. Pharm. Des. 31 (13), 1002–1012. 10.2174/0113816128402718250806151308 40814874

[B22] Jang J. E. Hajdu C. H. Liot C. Miller G. Dustin M. L. Bar-Sagi D. (2017). Crosstalk between regulatory T cells and tumor-associated dendritic cells negates anti-tumor immunity in pancreatic cancer. Cell Rep. 20 (3), 558–571. 10.1016/j.celrep.2017.06.062 28723561 PMC5649374

[B23] Kolli K. Kumar D. (2026). Scaffold optimization trends in matrix metalloproteinase inhibitors for selective pancreatic cancer Therapy-a review. Eur. J. Med. Chem. 305, 118540. 10.1016/j.ejmech.2025.118540 41539274

[B24] Li J. Che M. Zhang B. Zhao K. Wan C. Yang K. (2023). The association between the neuroendocrine system and the tumor immune microenvironment: emerging directions for cancer immunotherapy. Biochim. Biophys. Acta Rev. Cancer 1878 (6), 189007. 10.1016/j.bbcan.2023.189007 37907132

[B25] Li T. Hu C. Huang T. Zhou Y. Tian Q. Chen H. (2025). Cancer-associated fibroblasts foster a high-lactate microenvironment to drive perineural invasion in pancreatic cancer. Cancer Res. 85 (12), 2199–2217. 10.1158/0008-5472.Can-24-3173 40138590 PMC12167935

[B26] Liang J. Zhu W. Pan R. Wei S. Zhang S. Zhang Z. (2026). Spatiotemporal dynamic regulation of the Cx3cl1-Cx3cr1 axis: a double-edged sword in the tumor immune microenvironment and new strategies for precision therapy. Cytokine Growth Factor Rev. 88, 67–79. 10.1016/j.cytogfr.2026.01.006 41564496

[B27] Liao F. Tong Y. Sun H. Chen S. Wen S. Du Y. E. (2026). Nerves stimulates crosstalk between gastric cancer and group 3 innate lymphoid cells to enhance immunosuppression. Cancer Res. 10.1158/0008-5472.Can-25-3092 41534088 PMC13080324

[B28] Liu J. Ma X. Hu X. Wen J. Zhang H. Xu J. (2023). Schwann cell-specific rhoa knockout accelerates peripheral nerve regeneration *via* promoting schwann cell dedifferentiation. Glia 71 (7), 1715–1728. 10.1002/glia.24365 36971019

[B29] Liu Q. Q. Dong Z. K. Wang Y. F. Jin W. L. (2025). Reprogramming neural-tumor crosstalk: emerging therapeutic dimensions and targeting strategies. Mil. Med. Res. 12 (1), 73. 10.1186/s40779-025-00661-9 41163091 PMC12574003

[B30] Maietta I. Arístegui P. D. Álvarez I. G. Atanes O. R. García Fontán E. M. Bautista S. T. (2025). Sequential Yap1/Fosl1 silencing and epigenetic therapy to overcome stromal barriers in pancreatic cancer. Int. J. Pharm. 684, 126155. 10.1016/j.ijpharm.2025.126155 40925515

[B31] Marcadis A. R. Kao E. Wang Q. Chen C. H. Gusain L. Powers A. (2023). Rapid cancer cell perineural invasion utilizes amoeboid migration. Proc. Natl. Acad. Sci. U. S. A. 120 (17), e2210735120. 10.1073/pnas.2210735120 37075074 PMC10151474

[B32] Meng S. Hara T. Sato H. Tatekawa S. Tsuji Y. Saito Y. (2024). Revealing neuropilin expression patterns in pancreatic cancer: from single-cell to therapeutic opportunities (Review). Oncol. Lett. 27(3), 113. 10.3892/ol.2024.14247 38304169 PMC10831399

[B33] Niu Y. Förster S. Muders M. (2022). The role of perineural invasion in prostate cancer and its prognostic significance. Cancers (Basel) 14 (17), 4065. 10.3390/cancers14174065 36077602 PMC9454778

[B34] Nozzoli F. Catalano M. Messerini L. Cianchi F. Nassini R. De Logu F. (2024). Perineural invasion score system and clinical outcomes in resected pancreatic cancer patients. Pancreatology 24 (4), 553–561. 10.1016/j.pan.2024.03.004 38514359

[B35] Pantziarka P. Bouche G. Sukhatme V. Meheus L. Rooman I. Sukhatme V. P. (2016). Repurposing drugs in oncology (Redo)-Propranolol as an anti-cancer agent. Ecancermedicalscience 10, 680. 10.3332/ecancer.2016.680 27899953 PMC5102691

[B36] Partecke L. I. Speerforck S. Käding A. Seubert F. Kühn S. Lorenz E. (2016). Chronic stress increases experimental pancreatic cancer growth, reduces survival and can be antagonised by beta-adrenergic receptor blockade. Pancreatology 16 (3), 423–433. 10.1016/j.pan.2016.03.005 27083074

[B37] Perusina L. M. Zhang Y. Girgis A. Kasselman S. Lazarus J. Kryczek I. (2020). Interleukin 22 signaling regulates acinar cell plasticity to promote pancreatic tumor development in mice. Gastroenterology 158 (5), 1417–1432.e11. 10.1053/j.gastro.2019.12.010 31843590 PMC7197347

[B38] Pu N. Bo X. Lu H. Chen F. Zhou Y. Cheng Q. (2025). Fosl1: the core regulatory hub of tumor-neural interactions and its clinical translational prospects. Int. Immunopharmacol. 166, 115645. 10.1016/j.intimp.2025.115645 41056772

[B39] Qian X. Liu E. Zhang C. Feng R. Tran N. Zhai W. (2024). Promotion of perineural invasion of cholangiocarcinoma by schwann cells Via nerve growth factor. J. Gastrointest. Oncol. 15 (3), 1198–1213. 10.21037/jgo-24-309 38989424 PMC11231841

[B40] Ren L. Liu C. Çifcibaşı K. Ballmann M. Rammes G. Mota Reyes C. (2025). Sensory neurons drive pancreatic cancer progression through glutamatergic neuron-cancer pseudo-synapses. Cancer Cell 43 (12), 2241–2258.e8. 10.1016/j.ccell.2025.09.003 41005304

[B41] Scheff N. N. Saloman J. L. (2021). Neuroimmunology of cancer and associated symptomology. Immunol. Cell Biol. 99 (9), 949–961. 10.1111/imcb.12496 34355434 PMC9250294

[B42] Sharawy I. (2022). Neuroimmune crosstalk and its impact on cancer therapy and research. Discov. Oncol. 13 (1), 80. 10.1007/s12672-022-00547-5 35997976 PMC9399329

[B43] Steele C. W. Karim S. A. Leach J. D. G. Bailey P. Upstill-Goddard R. Rishi L. (2016). Cxcr2 inhibition profoundly suppresses metastases and augments immunotherapy in pancreatic ductal adenocarcinoma. Cancer Cell 29 (6), 832–845. 10.1016/j.ccell.2016.04.014 27265504 PMC4912354

[B44] Stoop T. F. Javed A. A. Oba A. Koerkamp B. G. Seufferlein T. Wilmink J. W. (2025). Pancreatic cancer. Lancet 405 (10485), 1182–1202. 10.1016/s0140-6736(25)00261-2 40187844

[B45] Sun Z. Xi Y. Lan X. (2025). Conceptual quantitative systems pharmacology framework for supporting clinical trial design in organ-specific autoimmune and rare diseases. Front. Immunol. 16, 1714438. 10.3389/fimmu.2025.1714438 41459511 PMC12741140

[B46] Sun L. Wang J. Zhang L. (2026). The role of sphingomyelin synthase 2 in lipid metabolism and its implications in diseases. Cell Biol. Int. 50 (1), e70103. 10.1002/cbin.70103 41268620

[B47] Tcyganov E. Mastio J. Chen E. Gabrilovich D. I. (2018). Plasticity of myeloid-derived suppressor cells in cancer. Curr. Opin. Immunol. 51, 76–82. 10.1016/j.coi.2018.03.009 29547768 PMC5943174

[B48] Tsuboi J. Eguchi A. Inoue H. Ichishi M. Go M. Okajima R. (2025). Plexin domain containing 2, a protein specifically expressed and elevated in human pancreatic cancer tissue and serum, influences cell proliferation by correlating with cortactin. Cancer Med. 14 (23), e71459. 10.1002/cam4.71459 41365610 PMC12688484

[B49] Udumyan R. Montgomery S. Fang F. Almroth H. Valdimarsdottir U. Ekbom A. (2017). Beta-blocker drug use and survival among patients with pancreatic adenocarcinoma. Cancer Res. 77 (13), 3700–3707. 10.1158/0008-5472.Can-17-0108 28473530

[B50] Vanden Abeele F. Salzet M. (2025). The neuro-immune oncology axis. Cancer Lett. 634, 218070. 10.1016/j.canlet.2025.218070 41033608

[B51] Wakiya T. Ishido K. Kimura N. Nagase H. Yoshizawa T. Morohashi S. (2021). Eukaryotic initiation factor 2 signaling behind neural invasion linked with lymphatic and vascular invasion in pancreatic cancer. Sci. Rep. 11 (1), 21197. 10.1038/s41598-021-00727-3 34707166 PMC8551178

[B52] Wang H. Zheng Q. Lu Z. Wang L. Ding L. Xia L. (2021). Role of the nervous system in cancers: a review. Cell Death Discov. 7 (1), 76. 10.1038/s41420-021-00450-y 33846291 PMC8041826

[B53] Wang L. Ge J. Han H. Jia Y. Qin Y. (2024). Crosstalk between the nervous system and tumor microenvironment: functional aspects and potential therapeutic strategies. Cancer Lett. 594, 216986. 10.1016/j.canlet.2024.216986 38797233

[B54] Wang Y. Ye Z. Yuan Y. Wang C. Chen G. Zhang Y. (2025). Sensory neuro-tumor crosstalk: therapeutic opportunities and emerging frontiers in cancer neuroscience. Biochim. Biophys. Acta Rev. Cancer 1880 (6), 189464. 10.1016/j.bbcan.2025.189464 41016671

[B55] Wang K. Ni B. Xie Y. Li Z. Yuan L. Meng C. (2025). Nociceptor neurons promote pdac progression and cancer pain by interaction with cancer-associated fibroblasts and suppression of natural killer cells. Cell Res. 35 (5), 362–380. 10.1038/s41422-025-01098-4 40122998 PMC12012126

[B56] Wang X. Yan Y. Li L. Li T. Thakur A. Zhang K. (2026). The influence of neuro-tumor interactions on tumorigenesis and therapeutic response. Exp. Hematol. Oncol. 15 (1), 18. 10.1186/s40164-026-00752-w 41645346 PMC12882464

[B57] Wong F. C. Merker S. R. Bauer L. Han Y. Le V. M. H. Wenzel C. (2025). Extracellular vesicles from pancreatic cancer and its tumour microenvironment promote increased schwann cell migration. Br. J. Cancer 132 (4), 326–339. 10.1038/s41416-024-02915-0 39863771 PMC11832759

[B58] Wu S. Xie L. Lin S. X. Wirth G. J. Lu M. Zhang Y. (2020). Quantification of perineural invasion focus after radical prostatectomy could improve predictive power of recurrence. Hum. Pathol. 104, 96–104. 10.1016/j.humpath.2020.07.005 32673683

[B59] Xie W. Xu J. Lu S. Zhang Y. (2024). Current therapeutic landscape and resistance mechanisms to larotrectinib. Cancer Biol. Med. 20 (12), 967–971. 10.20892/j.issn.2095-3941.2023.0471 38318928 PMC10845932

[B60] Xu J. Lou S. Huang H. Xu J. Luo F. (2025a). Regulation and crosstalk of cells and factors in the pancreatic cancer microenvironment. Curr. Cancer Drug Targets 25 (9), 1029–1048. 10.2174/0115680096317840240723071018 39225222

[B61] Xu M. Yu Y. Cao C. (2025). Cgrp-mediated neural addiction in tumor dynamic remodeling. Trends Cancer 11 (9), 834–838. 10.1016/j.trecan.2025.07.004 40769818

[B62] Xu N. Bian S. Lyu P. He X. Zheng W. (2025). Dynamic interplay of neuroendocrine signaling and immuno-surveillance in tumor niche remodeling. Crit. Rev. Oncol. Hematol. 216, 104958. 10.1016/j.critrevonc.2025.104958 40975450

[B63] Xu J. Yao H. Wang J. Jin Y. Chang W. Li L. (2025b). Perineural invasion and the “cold” tumor microenvironment in pancreatic cancer: mechanisms of crosstalk and therapeutic opportunities. Front. Immunol. 16, 1650117. 10.3389/fimmu.2025.1650117 40909268 PMC12404959

[B64] Zhang X. Lu H. Hong W. Liu L. Wang S. Zhou M. (2018). Tyrphostin B42 attenuates trichostatin a-Mediated resistance in pancreatic cancer cells by antagonizing Il-6/Jak2/Stat3 signaling. Oncol. Rep. 39 (4), 1892–1900. 10.3892/or.2018.6241 29393444

[B65] Zhang C. Guo X. Liu P. Wang Y. Zhang J. Li L. (2025). The neuro-immune axis in cancer: from mechanisms to therapeutic opportunities. J. Hematol. Oncol. 18 (1), 93. 10.1186/s13045-025-01748-5 41152924 PMC12570465

[B66] Zhang F. Wang Y. Liu F. Li Y. Liu X. Ren X. (2025). Impact of beta blockers on cancer neuroimmunology: a systematic review and meta-analysis of survival outcomes and immune modulation. Front. Immunol. 16, 1635331. 10.3389/fimmu.2025.1635331 40842986 PMC12364651

[B67] Zhang M. Liu N. Asam K. Meng C. Aouizerat B. Ye Y. (2025). Tnfr1 suppression by Xpro1595 reduces peripheral neuropathies associated with perineural invasion in female mice. Cells 14 (22), 1749. 10.3390/cells14221749 41294802 PMC12651030

[B68] Zhang S. Yuan L. Lin P. Yang G. Zhou X. Xu J. (2026). Cancer neuroscience: signaling pathways and new therapeutic strategies for cancer. Signal Transduct. Target Ther. 11 (1), 66. 10.1038/s41392-025-02364-y 41724757 PMC12926231

[B69] Zhao J. W. Zhu J. Y. Yang Z. Zhai Y. Y. Zhao C. Lu Z. (2025). Extracellular vesicle-mediated O-Glcnacase transfer drives neuronal necroptosis to facilitate gallbladder cancer perineural invasion. Cancer Res. 86 (6), 1392–1413. 10.1158/0008-5472.Can-25-2237 41460724 PMC13012253

[B70] Zu Z. Zhang C. Shi J. Chen K. Tang H. Hu K. (2025). Single-cell analysis reveals that Gfap(+) dedifferentiated Schwann cells promote tumor progress in Pni-Positive distal cholangiocarcinoma Via Lactate/Hmgb1 axis. Cell Death Dis. 16 (1), 215. 10.1038/s41419-025-07543-x 40148311 PMC11950304

